# Genetic and Epigenetic Association of Hepatocyte Nuclear Factor-1α with Glycosylation in Post-Traumatic Stress Disorder

**DOI:** 10.3390/genes13061063

**Published:** 2022-06-14

**Authors:** Lucija Tudor, Marcela Konjevod, Gordana Nedic Erjavec, Matea Nikolac Perkovic, Suzana Uzun, Oliver Kozumplik, Vlatka Zoldos, Gordan Lauc, Dubravka Svob Strac, Nela Pivac

**Affiliations:** 1 Laboratory for Molecular Neuropsychiatry, Division of Molecular Medicine, Ruder Boskovic Institute, 10000 Zagreb, Croatia; lucija.tudor@irb.hr (L.T.); marcela.konjevod@irb.hr (M.K.); gordana.nedic.erjavec@irb.hr (G.N.E.); matea.nikolac.perkovic@irb.hr (M.N.P.); dsvob@irb.hr (D.S.S.); 2 Department for Biological Psychiatry and Psychogeriatrics, University Hospital Vrapce, 10000 Zagreb, Croatia; suzana.uzun@gmail.com (S.U.); okozumplik@hotmail.com (O.K.); 3 School of Medicine, University of Zagreb, 10000 Zagreb, Croatia; 4 Faculty of Education and Rehabilitation Sciences, University of Zagreb, 10000 Zagreb, Croatia; 5 Department of Biology, Division of Molecular Biology, Faculty of Science, University of Zagreb, 10000 Zagreb, Croatia; vzoldos@biol.pmf.hr; 6 Glycobiology Laboratory, Genos Ltd., 10000 Zagreb, Croatia; glauc@genos.hr

**Keywords:** post-traumatic stress disorder, HNF1A, glycomics, N-glycans, polymorphism, methylation, inflammation

## Abstract

Post-traumatic stress disorder (PTSD) is a complex trauma-related disorder, the etiology and underlying molecular mechanisms of which are still unclear and probably involve different (epi)genetic and environmental factors. Protein N-glycosylation is a common post-translational modification that has been associated with several pathophysiological states, including inflammation and PTSD. Hepatocyte nuclear factor-1α (HNF1A) is a transcriptional regulator of many genes involved in the inflammatory processes, and it has been identified as master regulator of plasma protein glycosylation. The aim of this study was to determine the association between N-glycan levels in plasma and immunoglobulin G, methylation at four CpG positions in the *HNF1A* gene, *HNF1A* antisense RNA 1 (*HNF1A-AS1*), rs7953249 and *HNF1A* rs735396 polymorphisms in a total of 555 PTSD and control subjects. We found significant association of rs7953249 and rs735396 polymorphisms, as well as *HNF1A* gene methylation at the CpG3 site, with highly branched, galactosylated and sialyated plasma N-glycans, mostly in patients with PTSD. *HNF1A-AS1* rs7953249 polymorphism was also associated with PTSD; however, none of the polymorphisms were associated with *HNF1A* gene methylation. These results indicate a possible regulatory role of the investigated *HNF1A* polymorphisms with respect to the abundance of complex plasma N-glycans previously associated with proinflammatory response, which could contribute to the clinical manifestation of PTSD and its comorbidities.

## 1. Introduction

Post-traumatic stress disorder (PTSD) is a complex, trauma- and stressor-related disorder that develops in a subset of individuals as a result of direct or indirect exposure to a stressful or traumatic event(s) [1,2]. It is characterized by typical clusters of symptoms that include intrusive memories and re-experiencing, persistent avoidance, negative alterations in cognition and mood and changes in both physical and emotional reactions, including elevated arousal and reactivity [3]. In addition, PTSD is associated with an increased risk of developing various somatic diseases, including cardiovascular, autoimmune and inflammatory diseases [4,5]. However, the etiology and molecular mechanisms underlying PTSD are still unknown and represent challenging healthcare and scientific issues [6] arising from the interaction between different (epi)genetic and environmental factors. Various studies [6,7,8] have indicated significant involvement of genetic and epigenetic factors in PTSD development. Therefore, gene candidates and corresponding posttranslational modifications should be investigated in more detail in order to elucidate the complex biological underpinnings of PTSD.

One of the most common post-translational modifications is protein glycosylation, which can significantly alter the biological role of proteins and could represent a link between genetic inheritance and environmental stimuli [9]. N-glycosylation influences both the structure and function of proteins and plays an important role in several biological processes, including molecular trafficking, receptor activation, cell signaling and adhesion [10]. Although there are more than 700 different enzymes included in the synthesis and metabolism of glycans and their binding to proteins [11], there is no predefined pattern of protein glycosylation, which can be influenced by various intracellular changes, including gene expression, localization of sugar precursors and enzymes, as well as external stimuli [12]. Different pathophysiological states, such as inflammation, autoimmune diseases and mental disorders, including PTSD, have been associated with altered N-glycome [13]. This is in accordance with previous findings by Moreno-Villanueva and colleagues [5], who reported N-glycosylation changes related to accelerated aging in the patients with PTSD in comparison to the control group. Moreover, a recent study showed significant alterations in several plasma N-glycan levels, mostly tri- and tetra-antennary, highly galactosylated and sialyated N-glycans, in the patients with PTSD compared to healthy control subjects [1]. Changes in the N-glycan profile have also been associated with schizophrenia [14,15] and major depressive disorder [16], suggesting that PTSD shares similar biological and molecular foundations with other neuropsychiatric disorders that are characterized by the acute or chronic inflammation. In addition to neuropsychiatric disorders, the dysregulation of glycosylation and alterations in N-glycans have been associated with infectious diseases, aging, cancer, diabetes, cardiovascular, metabolic and other disorders [1,13,17,18].

Hepatocyte nuclear factor-1α (HNF1A) is a transcriptional regulator of many genes involved in the inflammatory response, and it has been identified as possible regulator of plasma protein glycosylation, in particular, glycan branching and plasma protein fucosylation [19,20]. Two polymorphisms in the *HNF1A* gene (rs7953249 and rs735396) were reported as significantly associated with protein glycosylation in the first GWAS study of human N-glycome [19]. The role of HNF1A in the regulation of expression of various genes, such as genes for fucosyltransferase, fucokinase, GDP-mannose-4,6-dehydratase, glucose transporter 2, pyruvate kinase and insulin, was confirmed in a recent study [21]. Moreover, some studies have reported a strong epigenetic influence of environmental factors on DNA methylation in the *HNF1A* gene [20], which was associated with certain alterations in plasma protein glycosylation [18,20,21]. Specifically, the altered methylation of four adjacent CpG sites located in the first exon of the *HNF1A* gene resulted in a significant change of the *HNF1A* RNA transcript levels in several cell lines [18,20]. In addition, changes in *HNF1A* methylation at CpG sites +172, +175, +178 and +182 from the exon 1 transcription start site showed association with the levels of the highly-branched N-glycans in the plasma [18], whereas *HNF1A* upregulation by targeted demethylation of these four CpG sites led to a decrease in complex, core-fucosylated N-glycans [20]. These findings suggest a regulatory role of cytosine methylation in the first exon of the *HNF1A* gene in its expression and glycosylation [18,20].

The aim of this study was to determine the association of rs7953249 polymorphism, located in the *HNF1A* antisense RNA 1 gene (*HNF1A-AS1*), and rs735396 polymorphism, located in intron 9 of the *HNF1A* gene, and their haplotype block with the levels of plasma and immunoglobulin G (IgG) N-glycans in a sample of 555 male patients, including PTSD patients and healthy individuals. Additionally, methylation of four CpG islands located in the *HNF1A* gene at positions +172, +175, +178 and +182 from the exon 1 transcription start site was determined in a subset of PTSD and control subjects and further analyzed in relation to investigated polymorphisms and levels of plasma and IgG N-glycans.

## 2. Materials and Methods

### 2.1. Participants

The study included 555 male subjects (258 patients with combat-related PTSD and 297 age-matched control subjects not exposed to combat trauma) recruited at the University Psychiatric Hospital Vrapce, Zagreb, Croatia, from October 2015 to February 2017. Individuals with current and chronic PTSD were diagnosed using a structured clinical interview (SCID) based on DSM-5 criteria [22], whereas the severity of PTSD was assessed using the Clinician-Administered PTSD Scale (CAPS) [23]. According to the frequency and intensity of the cluster symptoms (re-experiencing, avoidance and hyperarousal), the severity of PTSD symptoms can be divided into mild (CAPS scores: 46–65), moderate (CAPS scores: 66–95) and severe (CAPS scores: more than 95) [23]. The veterans with PTSD were unrelated Caucasian subjects of Croatian origin with an average age of 55 years (51; 61) and an average CAPS score of 86 (78; 88), indicating moderate PTSD symptom severity in most patients. For 30 days prior to blood sampling, the individuals with PTSD had not received any psychopharmacological therapy. Age-matched control subjects with a median age of 55 years (48; 62) were also evaluated using the same diagnostic instruments. Except for differences in the diagnosis of PTSD, the two experimental groups were subject to the same exclusion criteria: chronic drug abuse, alcohol dependence or pathophysiological changes in the liver, depression, schizophrenia, bipolar disorder, adult ADHD, Alzheimer’s disease (according to DSM-5 criteria), current or recent (previous 3 months) use of lipid-lowering agents, antihypertensive and antidiabetic medication. All participants were evaluated according to the International Classification of Diseases (ICD-10) to exclude patients with potential somatic diseases, such as fibrosis, sclerosis, cirrhosis and malignant liver disease. The study was approved by the Ethics Committee of the University Psychiatric Hospital Vrapce, Zagreb, Croatia, and the Bioethics Committee of the Ruder Boskovic Institute, Zagreb, Croatia, and all subjects signed an informed consent prior to initiation of the study procedures. Therefore, the study was conducted in accordance with the Declaration of Helsinki (1975) and its revised 2013 version.

### 2.2. Blood Processing

Blood samples (8.0 mL) from the subjects were collected in the morning after overnight fasting using BD Vacutainer™ glass blood collection tubes (Becton, Dickinson and Company, Franklin Lakes, NJ, USA) with 1.5 mL acid citrate dextrose (ACD) anticoagulant. The blood was processed on the same day; platelet-poor plasma was isolated via series of centrifugation (3 min at 3000× *g* followed by 15 min at 5000× *g*) as described previously [1]. DNA was extracted from peripheral blood using a salting-out method [24]. Plasma samples were immediately frozen and stored at −80 °C in 300 µL aliquots. Plasma samples, shipped on dry ice, were transferred for determination of plasma and IgG N-glycans. DNA samples were stored at +4 °C until further analyses.

### 2.3. N-glycan Determination in Plasma and IgG

The total plasma and IgG N-glycome of the platelet-poor plasma were determined using hydrophilic interaction high-performance liquid chromatography (HILIC) for plasma N-glycans [25] and ultraperformance liquid chromatography (UPLC) for IgG N-glycans after isolation of IgG with the affinity chromatography [26], as described previously.

### 2.4. IgG Isolation

The plasma samples (10× diluted) were applied to a protein G plate and filtered for approximately 5 min. After washing the plate 5 times with 5 column volumes (CV) of binding buffer to remove unbound proteins, IgG was released from the protein G monoliths using 5 CV of 1 M formic acid (pH 2.5) as elution solvent. Eluates were then collected and neutralized with 1 M ammonium bicarbonate to pH 7.0. Each step of the chromatographic procedure was conducted under vacuum conditions. The purity of the isolated IgG was verified by SDS-PAGE with NuPAGE Novex 4–12% Bis-Tris gels in an Xcell SureLock Mini-Cell (Invitrogen) according to the manufacturer’s instructions.

### 2.5. N-glycan Release and Labelling

Protein denaturation from plasma and IgG samples was performed with 2% (*w*/*v*) SDS (Invitrogen, Camarillo, CA, USA) for 10 min at 65 °C, followed by the addition of 4% (*v*/*v*) Igepal CA630 (Sigma Aldrich, St. Louis, MO, USA). N-glycans were released from the proteins by adding 1.2 U PNGase F (Promega, San Luis Obispo, CA, USA) and incubating overnight at 37 °C. Following extraction, N-glycans were fluorescently labeled with 2-aminobenzamide (2-AB) (Sigma Aldrich, St. Louis, MO, USA) after 2 h incubation at 65 °C.

### 2.6. Hydrophilic Interaction High-Performance Liquid Chromatography (HILIC)

The released fluorescently labeled plasma N-glycans were subjected to HILIC with an Acquity UPLC instrument (Waters, Milford, MA, USA) on a Waters BEH glycan chromatography column (150 × 2.1 mm i.d., 1.7 μm BEH particles) at 25 °C with 100 mM ammonium formate adjusted to pH 4.4 (solvent A) and acetonitrile (solvent B). Runs were performed using a linear gradient of solvent A (30–47%) at a 0.56 mL/min flow rate for 23 min and a fluorescence detector with excitation and emission wavelengths of 250 nm and 428 nm, respectively. The system was calibrated using an external standard of hydrolyzed and 2-AB-labeled glucose oligomers, from which the retention times for the individual N-glycans were converted to glucose units. The obtained chromatograms were separated into 39 chromatographic peaks that were previously assigned to each of the N-glycan species [17,27] (Supplementary Table S1). The amount of N-glycans present in each peak was expressed as percent of the total integrated chromatographic area using an automatic method with a traditional integration algorithm, followed by manual correction to maintain the same intervals of integration for all the samples.

### 2.7. Ultraperformance Liquid Chromatographic (UPLC) Analysis of IgG N-glycans

The fluorescently labelled IgG N-glycans were separated by UPLC on a Waters Acquity UPLC instrument (100 × 2.1 mm i.d., 1.7 μm BEH particles), with 100 mM ammonium formate (pH 4.4) as solvent A and acetonitrile as solvent B. Separation was performed using a linear gradient of 75–62% acetonitrile at a flow rate of 0.4 mL/min in a 20 min analytical run and an FLR fluorescence detector with excitation and emission wavelengths of 330 and 420 nm, respectively. The hydrolyzed and 2-AB-labeled glucose oligomers were used as external standard from, and the retention times for the individual N-glycans were converted to glucose units. The obtained chromatograms were separated into 24 peaks representing the percentage of the total integrated area. N-glycan species in each N-glycan peak (IgGP) had been previously assigned [26,28] (Supplementary Table S2).

### 2.8. Genotyping

The concentration and purity of isolated DNA was measured on an Implen™ NanoPhotometer™ N60 Micro-Volume UV/Vis spectrophotometer (Thermo Fisher Scientific, Waltham, MA, USA). *HNF1A-AS1* rs7953249 and *HNF1A* rs735396 polymorphisms were determined with a TaqMan genotyping assay (Thermo Fisher Scientific; Assay ID: C__29305540_10) and a KASP Genotyping Assay on Demand (LGC, Biosearch Technologies; Catalog No: KBS-2000-100), respectively, on an Applied Biosystems R 7300 real-time PCR system following the manufacturer’s protocol. The 10 µL reaction volume contained around 20 ng of DNA. Thermocycler conditions for TaqMan genotyping assays were as follows: 10 min at 95 °C (initial denaturation), 40 cycles of 95 °C for 15 s and 60 °C for 1 min. The conditions for KASP genotyping assays were 15 min at 94 °C (initial denaturation), 10 cycles of 94 °C for 20 s, 61–55 °C for 1 min (dropping 0.6 °C per cycle) and 26 cycles of 94 °C for 20 s and 55 °C for 1 min. Approximately 10% of randomly selected samples were genotyped again in order to control the quality of genotyping procedures, and a negative control (pure water) was included in each run.

### 2.9. Analysis of CpG Methylation in the HNF1A Gene

The methylation of the CpG sites in the *HNF1A* gene was analyzed using the DNA isolated from the peripheral nucleated cells in the whole blood samples of the 100 participants with PTSD and 100 control subjects. The methylation of the four CpG sites at +172 (CpG1), +175 (CpG2), +178 (CpG3) and +182 (CpG4) from the transcription start site in exon 1 of the *HNF1A* gene was analyzed using pyrosequencing as described previously [18]. Sodium bisulfite conversion was performed using an EZ DNA Methylation-Gold Kit (Zymo Research, Catalog No: D5007). With this method, sodium bisulfite converts unmethylated cytosines to uracils, whereas methylated cytosines remain unchanged. The bisulfite-treated DNA was eluted in 20 μL buffer at a final concentration of 25 ng/μL, after which a specific region in the *HNF1A* first exon was amplified using a PyroMark PCR kit (Qiagen, Catalog No: 978703) using the following primers: 5′-GGA TAA GGG GGA GTT TTG-3′; 5′-CCC CTC TAA ACT CTC CTA-3′ (biotinylated) [18]. A volume of 10 μL of the PCR products was sequenced using a PyroMark Q24 Advanced system according to the manufacturer’s protocol and using the pyrosequencing primer for *HNF1A* 5′-AAG GGG GAG TTT-3′ [18]. The fully methylated and unmethylated DNA controls, no-template control and built-in quality control for bisulfite treatment were incorporated in the analysis (Quigen, EpiTect Control DNA and Control DNA Set, Catalog No: 59695). Each target CpG site was evaluated as a T/C SNP and expressed as a percentage of methylated cytosines at each CpG position. Because the CpG4 site was almost fully methylated in all subjects, this CpG island was excluded from further analyses.

### 2.10. Statistical Analysis

To eliminate experimental variation from measurements, normalization and batch correction were performed on raw N-glycan data obtained by UPLC and expressed as percentages of the total area under the curve, as described previously [1]. Statistical analyses were performed using R Statistics 3.5.1. The normality of the distribution was assessed with the Kolmogorov–Smirnov test. Because the data distribution deviated from normal in the case of most N-glycan peaks, the results were expressed as median and interquartile range (25th and 75th percentile), and non-parametric analyses were performed. Multiple linear regression was used to determine the effect of age on the levels of plasma and IgG N-glycans. Because age was a significant predictor in this model and it is known that it affects the N-glycome [29], we corrected for the effect of age in the linear model of each N-glycan peak, and the obtained residuals were used for further statistical analysis. The subjects were subdivided according to different *HNF1A-AS1* rs7953249 and *HNF1A* rs735396 genotypes (genetic model) or alleles (allelic model), and the differences in the distribution of various N-glycans and methylation of *HNF1A* CpG sites were evaluated using a Kruskal–Wallis ANOVA on ranks and Mann–Whitney U test, respectively. Haplotype analysis for selected polymorphisms was performed using Haploview 4.2 software [30] to determine the linkage equilibrium (LD) values between these two SNPs based on the confidence interval method by Gabriel et al. [31]. Because obtained results showed strong LD (D’ = 0.81) between *HNF1A-AS1* rs7953249 and *HNF1A* rs735396 polymorphisms, PLINK 1.07 software was used to assign the most probable haplotype pairs to each subject using an expectation–maximization algorithm [32]. The Hardy–Weinberg (HW) equilibrium for tested SNPs was also calculated using Haploview 4.2. The differences in the frequencies of genotypes, alleles and haplotypes between the PTSD patients and control subjects were analyzed with an χ^2^-test. In the case of significant results, the standardized residuals (R) were calculated to determine which parameter contributed most to the significance. The correlations between N-glycans levels and methylation at each *HNF1A* CpG site were analyzed using Spearman’s rank test. Because associations of two *HNF1A* polymorphisms and three *HNF1A* CpG islands with N-glycan levels were analyzed, *p* value was set to 0.01.

## 3. Results

The genotyping results revealed that the minor allele frequency (MAF) of the *HNF1A-AS1* rs7953249 polymorphism (G allele) was 0.402 in all subjects (0.369 in patients with PTSD and 0.440 in control subjects), which is similar to the estimated MAF in the European population (0.440) [33]. Moreover, MAF of *HNF1A* rs735396 polymorphism (C allele) was 0.344 in all subjects (0.326 in PTSD patients and 0.364 in control subjects), which is in agreement with the estimated MAF in the European population (MAF = 0.380) [33]. The genotype distributions of both polymorphisms did not deviate from the Hardy–Weinberg equilibrium (χ^2^ = 0.005; df = 2; *p* = 0.997 for *HNF1A-AS1* rs7953249 polymorphism and χ^2^ = 0.057; df = 2; *p* = 0.972 for *HNF1A* rs735396 polymorphism).

**Figure 1:**
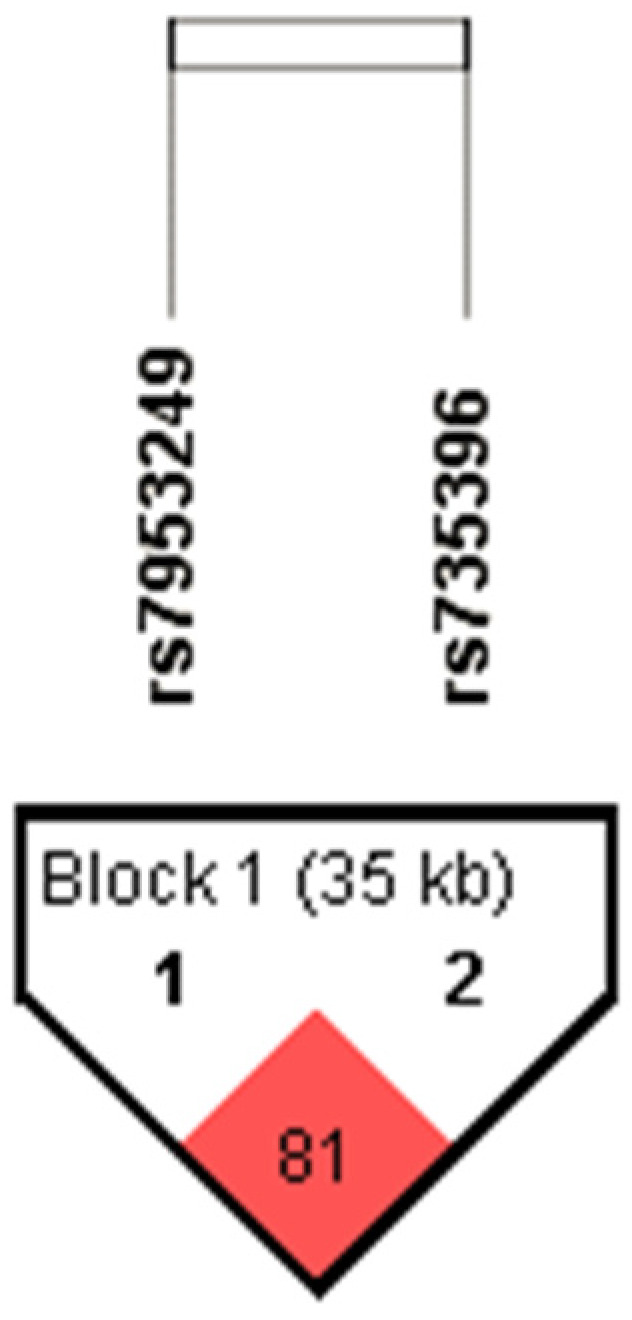
Linkage disequilibrium (LD) plot of two *HNF1A* polymorphisms (rs7953249 and rs735396). Pairwise LD value (D’ × 100) calculated for this SNP combination (LD = 81 as denoted in a red rectangle) indicates a strong link between rs7953249 and rs735396 polymorphisms.

In addition, a high degree of linkage disequilibrium (LD) was revealed between the polymorphisms *HNF1A-AS1* rs7953249 and *HNF1A* rs735396 (D’ = 0.81) (Figure 1). Therefore, in addition to genetic and allelic model, the haplotype pairs were assigned to the each subject using an expectation–maximization algorithm, and haplotype analysis was performed. The most common haplotype was AT (55.9%), followed by GC (30.5%). The least common haplotype pairs were GT (9.7%) and AC, which was detected in 3.9% of enrolled subjects.

### 3.1. Association of HNF1A Polymorphisms with PTSD

Significant differences were observed in the distribution of *HNF1A-AS1* rs7953249 genotypes. Specifically, the control subjects were more often carriers of the GG genotype (R = 2.0) compared to patients with PTSD (R = −1.9), (*p* = 0.010). Moreover, the frequency of A allele was higher in the PTSD patients than in the control subjects, although at a nominally significant level (*p* = 0.016) (Table 1). On the other hand, the distribution of *HNF1A* rs735396 genotypes (*p* = 0.061) and alleles (*p* = 0.208), as well as *HNF1A* rs7953249-rs735396 haplotypes (*p* = 0.094), was similar between the patients with PTSD and the control subjects (Table 1).

### 3.2. Association of HNF1A Gene Methylation and HNF1A Polymorphisms

**Figure 2:**
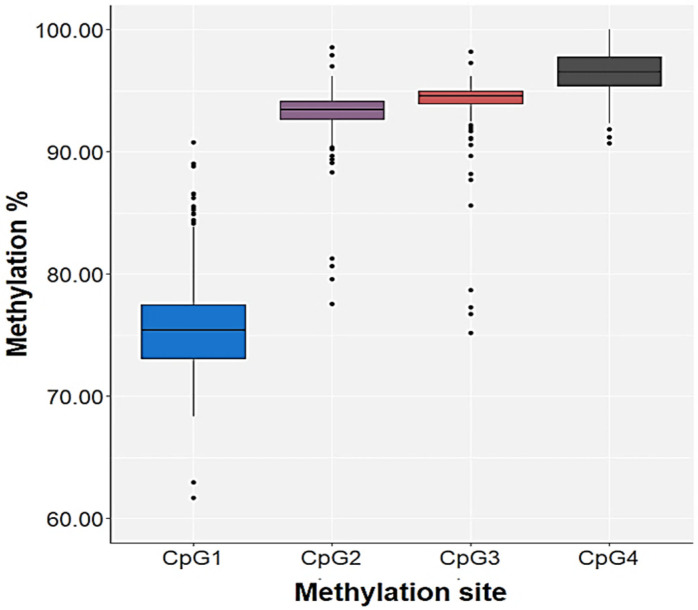
Methylation at four CpG sites of the *HNF1A* gene in all subjects. The central box represents the percentage of methylated cytosines (interquartile range), the middle line represents the median, the vertical line extends from the minimum to the maximum value and separate dots represent outliers.

To evaluate the association of CpG methylation in the *HNF1A* gene with the human plasma and IgG N-glycan levels, as well as to distinguish the possible differences in methylation of this gene coding for transcription factor between the patients with PTSD and control subjects, the four CpG sites in the *HNF1A* gene were analyzed: CpG1, located at +172; CpG2, located at +175; CpG3, located at +178; and CpG4, positioned at +182 from the transcription start site in exon 1 of *HNF1A* (NCBI Reference Sequence: NG_011731.2). The percentage of methylated cytosine at each of four CpG sites in the *HNF1A* gene for all subjects is presented in Figure 2.

Because the CpG4 site was nearly 100% methylated in all subjects, as similarly reported in a previous study by Zoldos et al. [18], this CpG position was excluded from further analysis. There were no significant differences in the methylation of CpG1 (U = 4353.5; *p* = 0.214), CpG2 (U = 4247.0; *p* = 0.131) or CpG3 (U = 4718.0; *p* = 0.740) sites between the patients with PTSD and control subjects; however, to check for an association between CpG methylation and *HNF1A* polymorphisms due to differences in the distribution of *HNF1A-AS1* rs7953249 genotypes between the two diagnostic groups, analysis was performed separately in the PTSD patients and control subjects. The differences in the methylation of 3 CpG sites were not associated with *HNF1A-AS1* rs7953249 or *HNF1A* rs735396 polymorphism or their haplotype in the control subjects nor in the subjects with PTSD (Table 2).

### 3.3. Correlation of HNF1A Gene Methylation and N-glycome

The correlation between the methylation at 3 CpG sites of the HNF1A gene and relative abundance of N-glycans in plasma and IgG was investigated separately in patients with PTSD and control subjects due to differences in N-glycome between the PTSD and control groups (data available upon request), which was also observed in our previous study [1].

Table 3 shows that N-glycans were significantly correlated with the percentage of methylated cytosine at the three CpG sites in the *HNF1A* gene; complete correlation data are available upon request. The most noticeable negative correlations were observed between the triantennary trigalactosylated plasma N-glycans (GP24 (*p* = 0.006), GP26 (*p* = 0.005), GP30 (*p* = 0.007) and GP31 (*p* = 0.010)) and CpG3 site methylation, whereas nominally negative correlations were found with CpG2 methylation (*p* < 0.05) in patients with PTSD but not in the control subjects. GP21 N-glycan was significantly negatively correlated with CpG1 methylation (*p* = 0.007), but its correlation with the CpG2 (*p* = 0.018) and CpG3 (*p* = 0.013) island methylation did not remain significant after correction for multiple testing. Biantennary GP10 N-glycan was significantly negatively correlated with the CpG2 site (*p* = 0.001), whereas GP14 N-glycan positively correlated with the CpG1 site (0.010) in control subjects. None of the IgG glycans, except IgGP14, which negatively correlated with CpG2 island methylation in control subjects, showed significant correlation with any of the investigated CpG sites (Table 3).

### 3.4. Association of HNF1A Polymorphisms and N-glycome

**Figure 3:**
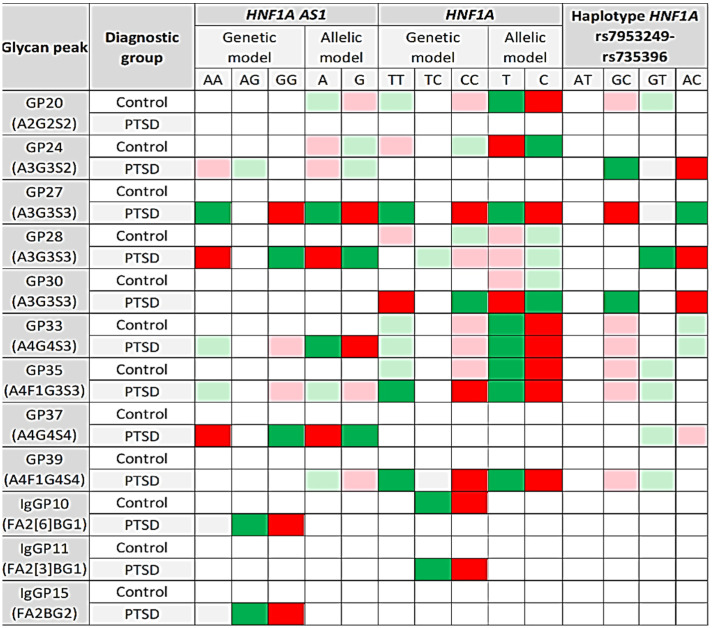
Summary of the significant (*p* < 0.010) and nominally significant (*p* < 0.050) changes in the N-glycan peaks associated with the *HNF1A-AS1* rs7953249 and *HNF1A* rs735396 polymorphisms and their haplotype block in PTSD and control subjects. Red cells represent significantly (intense red) or nominally significantly (light red) lower abundance of N-glycan structures in some genotypes, alleles or haplotypes. Green cells represent significantly (intense green), or nominally significantly (light green) higher abundance of N-glycan structures in some genotypes, alleles or haplotypes.

The association of the *HNF1A-AS1* rs7953249 and *HNF1A* rs735396 genotypes, alleles and haplotypes with plasma and IgG N-glycome was analyzed separately in the control subjects and PTSD patients. Table 4 and Supplementary Table S1 present the significant findings in both diagnostic groups. Full analysis data with all tested plasma and IgG glycan peaks are available upon request. Both SNPs showed the strongest association with the tri- and tetra-antennary galactosylated and sialyated plasma N-glycans in patients with PTSD, whereas less prominent association (nominally significant) or no association was found in the control group (Table 4, Figure 3, Supplementary Table S3). Most IgG N-glycans showed no association with these two SNPs; however, IgGP10 and IgGP15 N-glycans were significantly associated with the *HNF1A-AS1* rs7953249 polymorphism in the PTSD subjects (genetic model). In addition, IgGP10 and IgGP11 N-glycans demonstrated significant association with the *HNF1A* rs735396 polymorphism (genetic model) in the control, as well as in the PTSD group, respectively. All three IgG N-glycans significantly associated with the *HNF1A* polymorphisms are core-fucosylated, biantennary with bisecting galactose. Allelic and haplotypic analyses did not reveal significant associations with any of the IgG N-glycan peaks in the control subjects nor in patients with PTSD (Table 4, Figure 3, Supplementary Table S3).

**Figure 4:**
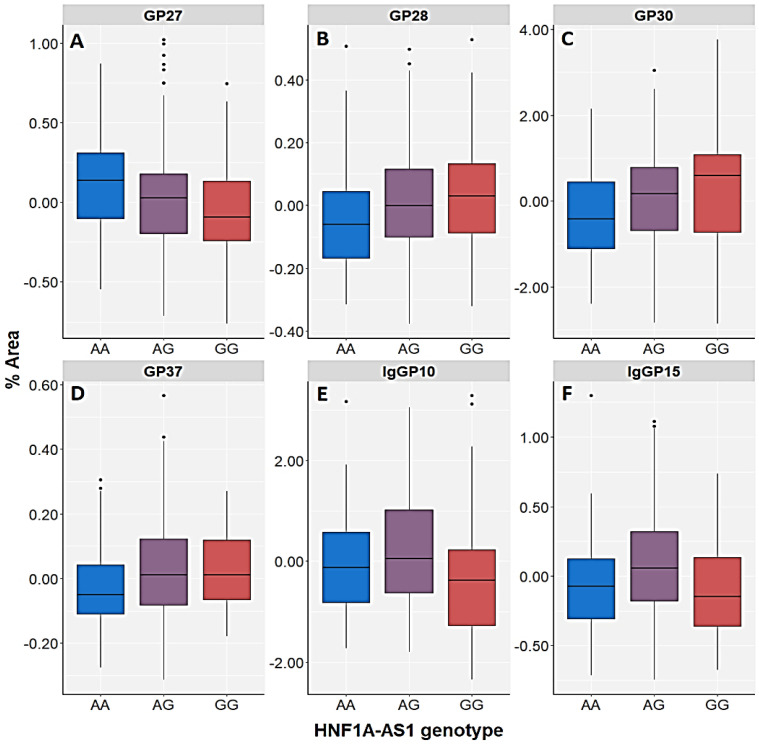
Relative distribution of the plasma N-glycan peaks (GP27 (A3G3S3) (**A**), GP28 (A3G3S3) (**B**), GP30 (A3G3S3) (**C**) and GP37 (A4G4S4) (**D**)), as well as the IgG N-glycan peaks (IgGP10 (FA2[6]BG1) (**E**) and IgGP15 (FA2BG2) (**F**)) in patients with PTSD subdivided according to different genotypes of the *HNF1A-AS1* rs7953249 polymorphism. The central box represents the interquartile range of the age-adjusted percentage of the total N-glycan peak area, the middle line represents the median, the vertical line extends from the minimum to the maximum value and separate dots represent outliers.

The AA homozygotes and A allele carriers of the *HNF1A-AS1* rs7953249 polymorphism had lower concentrations of GP28 (A3G3S3), GP30 (A3G3S3) and GP37 (A4G4S4) plasma N-glycans compared to GG homozygotes and G allele carriers in the group of patients with PTSD but not in the control group (Table 4, Figure 3 and Figure 4). A similar trend was observed regarding GP24 (A3G3S2) N-glycan, but at a nominally significant level (Figure 3, Supplementary Table S3). In contrast, higher levels of GP27, in which the dominant N-glycan type is also A3G3S3, were observed in PTSD subjects who were AA homozygotes (A allele carriers) compared to the GG homozygotes (G allele carriers) of the *HNF1A-AS1* rs7953249 polymorphism (Figure 4). This trend was also noticeable with the GP33 (A4G4S3) and GP35 (A4F1G3S3) N-glycans, but it did not remain significant after correction for multiple testing (Figure 3, Supplementary Table S3). The highest levels of both IgGP10 (FA2[6]BG1) and IgGP15 (FA2BG2) N-glycans had heterozygotes with PTSD, whereas GG homozygous PTSD patients had the lowest levels (Figure 4, Supplementary Table S1), although the association of the *HNF1A-AS1* rs7953249 polymorphism with IgG N-glycans was only detected in the genetic model (Table 4, Figure 3, Supplementary Table S3).

**Figure 5:**
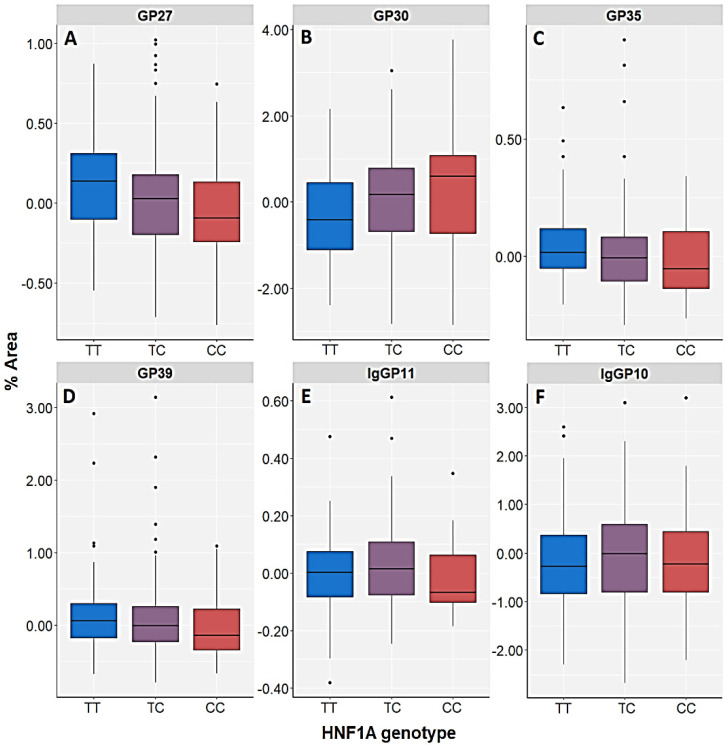
Relative distribution of the plasma N-glycan peaks (GP27 (A3G3S3) (**A**), GP30 (A3G3S3) (**B**), GP35 (A4F1G3S3) (**C**) and GP39 (A4F1G4S4) (**D**)), as well as the IgG N-glycan peak IgGP11 (FA2[3]BG1) (**E**) in patients with PTSD and the IgG N-glycan peak IgGP10 (FA2[6]BG1) (**F**) in the control subjects, both subdivided according to different genotypes of the *HNF1A* rs735396 polymorphism. The central box represents the interquartile range of the age-adjusted percentage of the total N-glycan peak area, the middle line represents the median, the vertical line extends from the minimum to the maximum value and separate dots represent outliers.

Furthermore, TT homozygotes of the *HNF1A* rs735396 polymorphism had the highest levels of the GP27 (A3G3S3), GP35 (A4F1G3S3) and GP39 (A4F1G4S4) N-glycans and nominally higher levels of GP33 (A4G4S3) N-glycan in the patients with PTSD, whereas CC homozygotes had the lowest levels (Table 4, Figure 3 and Figure 5). This trend was also observed in control subjects, whose TT carriers presented with higher levels of GP20 (A2G2S2), GP33 (A4G4S3) and GP35 (A4F1G3S3) N-glycans compared to CC homozygotes (nominal significance) (Figure 3, Supplementary Table S3). Likewise, higher levels of GP20, GP33 and GP35 N-glycans and nominally higher levels of GP27 N-glycan in the control subjects, as well as higher levels of GP27, GP33, GP35 and GP39 N-glycans in PTSD patients were associated with T allele of the *HNF1A* rs735396 polymorphism (Table 4, Figure 3, Supplementary Table S3).

In contrast, CC homozygotes and C allele carriers of the *HNF1A* rs735396 polymorphism presented with the highest levels of GP30 (A3G3S3) N-glycan in the PTSD subjects and nominally higher levels of GP24 (A3G3S2) and GP28 (A3G3S3) N-glycans in the control subjects (Figure 3 and Figure 5, Supplementary Table S3). Similarly to the *HNF1A-AS1* rs7953249 polymorphism, the heterozygotes of the *HNF1A* rs735396 polymorphism had the highest levels of IgGP11 (FA2[3]BG1) N-glycan in the PTSD group and the highest levels of IgGP10 (FA2[6]BG1) N-glycan in the control subjects (Figure 5, Supplementary Table S3).

Haplotype analysis of the *HNF1A-AS1* rs7953249 and the *HNF1A* rs735396 polymorphisms demonstrated a significant association of the rarest AC haplotype with the lower levels of GP24, GP28 and GP30 N-glycans and nominally significant association with the lower levels of GP37 N-glycan in the PTSD subjects (Table 4, Figure 3, Supplementary Table S3).

Moreover, a significant association of the rarest AC haplotype was observed with the higher levels of GP27 N-glycan, as well as a nominally significant association with the higher levels of GP33 N-glycan; however, no association was found with IgG N-glycans in any of the diagnostic groups (Table 4, Figure 3, Supplementary Table S3).

## 4. Discussion

The HNF1A and its downstream target protein, HNF4A, are both transcriptional factors, mostly expressed in the liver and pancreas, that form a complex cross-regulatory network through which they regulate the expression of many genes involved in various metabolic [34] and immunological processes [35,36]. The majority of the immunoglobulins and N-glycans are synthetized in the liver [37], and it is assumed that HNF1A and HNF4A regulate protein glycosylation. In particular, they stimulate antennary fucosylation by moderating the expression of antennary fucosyltransferases (FUT3, FUT5, FUT6), L-fucokinase and GDP-manose-4,6-dehydratase (GMDS) by downregulating the expression of α-(1,6)-fucosyltransferase (FUT8), the enzyme that adds fucose to the glycan core [19,21].

Mutations in the *HNF4A* and *HNF1A* genes are the cause of the most frequent type of types 1 and 3 maturity-onset diabetes of the young (HNF1A-MODY), respectively [38,39] and also represent a risk for type 2 diabetes [40], metabolic syndrome [41,42], coronary heart disease [43,44], pancreas and liver cancer [45,46] and ulcerative colitis [47]. Moreover, variations in the *HNF1A* gene have been associated with LDL-cholesterol [48], γ-glutamyl transferase [49], as well as C-reactive protein (CRP) levels in the plasma [35,36,41]. CRP is a proinflammatory marker that strongly predicts the development of cardiovascular and metabolic diseases, and it is also elevated in patients with PTSD [50,51,52], who are at greater risk of developing these comorbidities compared to healthy individuals [4]. Increased inflammation state of patients with PTSD is also reflected in altered N-glycome, in particular, higher levels of tri- and tetra-antennary galactosylated and sialyated N-glycan structures in the plasma [1]. Increased complexity of plasma N-glycans is also associated with other neuropsychiatric disorders [14,16], as well as diabetes [53], HNF1A-MODY [54] and other autoimmune and inflammation-related states [17,21].

In the first GWAS study that analyzed human N-glycome [19], the G allele of *HNF1A-AS1* rs7953249 polymorphism and the C allele of the *HNF1A* rs735396 polymorphism were associated with lower levels of antennary fucosylated glycan structures (A2F1G2), whereas the C allele of the *HNF1A* rs735396 polymorphism was also associated with levels of highly branched tetra-antennary N-glycans (A4G4 and FA4G4) [19]. Follow-up studies [55,56] confirmed the association of the rs735396 polymorphism with A2F1G2 N-glycan distribution and revealed additional SNPs located at 5′ end of the *HNF1A* gene associated with the levels of the tri and tetra-antennary glycans and antennary fucosylation. This is similar to the results obtained in our study, where we showed that the *HNF1A-AS1* rs7953249 and *HNF1A* rs735396 polymorphisms, as well as their haplotype, were mostly associated with the levels of the tri and tetra-antennary highly sialyated and galactosylated plasma N-glycans and, to a lesser extent, to the IgG N-glycans in subjects with PTSD. In our study, several N-glycans that share the same structures showed associations with the *HNF1A* polymorphisms but in a different direction. We observed that the G allele and/or GG genotype of the *HNF1A-AS1* rs7953249 polymorphism were associated with lower levels of triantennary glycans containing only an α2–3 bond (A3G3S(3,3,3)3), whereas the glycan structures with at least one α2–6 sialic bond (A3G3S(3,3,6)3, A4G4S(3,3,3,6)4) were presented with higher levels in the G carriers. Similar differences were observed for the *HNF1A* rs735396 SNP in our study, also mostly in the PTSD subjects. The T allele of the *HNF1A* rs735396 polymorphism was associated with higher levels of several plasma tri- and tetra-antennary N-glycans containing α2-3 bonds and lower levels of triantennary glycans containing α2–6 linked sialic acids.

Different enzymes are involved in the formation of these two linkage types [57], and inverse regulation of α2,3- and α2,6-sialylation has been reported in diabetes [58]. Specifically, biantennary α2,6-sialylation was increased, whereas triantennary α2,3-sialylation was decreased in individuals with type 2 diabetes compared to the control group [58]. For example, an SNP in *ST6GAL1*, a gene encoding the β-galactoside α2,6-sialyltransferase, was associated with type 2 diabetes predisposition in Southeast Asians in a recent GWAS study [59], supporting growing evidence of the role of ST6GAL1 in inflammation [60]. Therefore, our results indicate that besides moderating core and antennary fucosylation, HNF1A could also regulate the levels of highly branched sialyated N-glycans based on the type of linkage by which sialic acid is attached to the glycan.

The association of the *HNF1A-AS1* rs7953249 and the *HNF1A* rs735396 with IgG N-glycans was not as clear as with the plasma-derived N-glycans. The higher levels of the IgG N-glycan structures FA2[6]BG1, FA2[3]BG1, and FA2BG2 demonstrate the association with the *HNF1A-AS1* rs7953249 or the *HNF1A* rs735396 heterozygotes; however, it was only observed in the genetic model. All of these IgG glycan structures are core-fucosylated and galactosylated, which is usually related to r inflammation [61]; however, they could also be considered proinflammatory due to the presence of bisecting N-acetylglucosamine (GlcNAc) associated with enhanced antibody-dependent cellular cytotoxicity (ADCC) [61]. Without significant allelic associations with the levels of glycan structures of varying complexity, an unambiguous conclusion could not be drawn.

The functional role of the *HNF1A-AS1* rs7953249 polymorphism, located in the 5′ *HNF1A* gene region, and its effects on *HNF1A* expression are still unknow, but there have been reports of an association of rs7953249 SNP with ischemic brain injury, with the G allele demonstrating a protective effect against small vessel disease, a subtype of brain ischemia [62]. Similarly, in our study, the GG genotype of the *HNF1A-AS1* rs7953249 polymorphism was more prevalent among the control subjects compared to PTSD patients, indicating the potential protective role of the G allele in the development of PTSD. However, in patients with PTSD included in our study, this allele was also associated with a higher abundance of the N-glycan types, which are characteristic of inflammation.

The C allele of the *HNF1A* rs735396 polymorphism was previously associated with the components of metabolic syndrome in the Tunisian population [63], as well as altered CRP levels [35,36,41], whereas the T allele was associated with the development of pancreatic cancer [46]. In our study, no significant differences were detected in the distribution of the *HNF1A* rs735396 genotypes and alleles between the PTSD and control groups; however, this SNP was found to be indicative of increased inflammation based on the observed associations with plasma N-glycome in patients with PTSD. It has been shown that the *HNF1A* rs735396 polymorphism, occurring in intron 9, which is located in the enhancer regulatory region, might modulate *HNF1A* expression [46]. Specifically, the *HNF1A* rs735396 T allele was associated with lower expression of HNF1A protein in the Han Chinese population [46]. Based on these data, it could be suggested that lower expression of the HNF1A transcription factor, possibly influenced by the T allele of the *HNF1A* rs735396 polymorphism, might be associated with the abundance of highly branched and sialyated plasma N-glycans observed in our study.

However, we detected negative correlations between the levels of triantennary galactosylated and sialyated plasma N-glycans and CpG3 site methylation (and possibly higher *HNF1A* expression) in patients with PTSD, which indicates the opposite. Our results with respect to *HNF1A* methylation are in contrast with previous results demonstrating a positive association of *HNF1A* methylation with levels of highly-branched N-glycans in plasma [18]. Moreover, lower expression of *HNF1A* in pancreatic β cells was observed in mice and humans with diabetes [64], as well as in a pancreatic carcinoma cell line [46]; it is possible that *HNF1A* expression is partially regulated by CpG methylation of the *HNF1A* gene. In our study, none of the tested SNPs showed associations with *HNF1A* methylation in peripheral nucleated cells. However, it is possible that these polymorphisms regulate the methylation of the *HNF1A* gene and, consequently, its expression in other tissues, such as the liver, resulting in increased levels of inflammatory N-glycans in plasma. Induced global hypomethylation was reported to lead to significant changes in the abundance of core fucosylated and highly branched and sialyated plasma and IgG N-glycans [27,65]. However, although a high correlation of DNA methylation across different tissues has been reported [66], we did not observe associations of *HNF1A* methylation in the blood cells with the specific N-glycan species in the same direction. This is potentially due to a lower number of subjects for whom *HNF1A* methylation status was determined, as well as the inclusion of only male participants, whose glycosylation dynamics show differences in comparison to female subjects [67]. This may have led to the lower statistical power and contradictory results we observed. We also did not identify any prominent associations of IgG N-glycans with *HNF1A* methylation or HNF1A SNPs, similar to results reported in a previous study [18]. These findings could be explained by the fact that the majority of IgG N-glycans are biantennary and are rarely tri-, tetra-antennary or antennary fucosylated.

Most N-glycans in plasma are produced in the liver, with the exception of highly branched tetra-antennary N-glycans, which are probably produced by immune cells and are associated with inflammatory processes [68]. This could explain why most of the observed associations of these types of glycans with the *HNF1A* polymorphisms and *HNF1A* methylation were obtained in patients with PTSD and not in controls subjects. These findings also highlight the need to study plasma N-glycans as important indicators of systemic inflammation, although the mechanisms by which IgG N-glycans influence different pathophysiological states are more recognized. Plasma CRP levels were associated both with the *HNF1A* polymorphisms (including rs7953249 and rs735396) [35] and plasma N-glycan levels, especially those with trisialyated structures [69], which showed the highest association with *HNF1A* SNPs in our study.

These results indicate a possible regulatory role of the *HNF1A* rs7953249 and rs735396 polymorphisms and their haplotypes with respect to the abundance of complex plasma N-glycans and proinflammatory cytokines in patients with PTSD, possibly through mechanisms other than *HNF1A* methylation. The joint interaction between HNF1A, CRP, glycosylation and inflammation could underlie the biological background of many neuropsychiatric disorders, including PTSD, as well as their comorbid somatic diseases. However, N-glycome is significantly affected by age, sex, reproductive cycle, race and several metabolic parameters, which should be taken into account in analysis [67]. The association between epigenetic silencing of the *HNF1A* gene in peripheral nucleated cells and N-glycan branching could not be clearly elucidated due to the small sample size of analyzed subjects, as well as differential results regarding the association between *HNF1A* methylation and glycosylation in various tissues. In addition, the levels of the HNF1A in liver tissues and blood cells should be analyzed to determine the effects of the *HNF1A-AS1* rs7953249 and the *HNF1A* rs735396 polymorphisms on HNF1A expression.

In conclusion, to the best of our knowledge, this is the first association study to investigate the association of *HNF1A* polymorphisms, glycosylation and epigenetic regulation of the HNF1A gene in the blood and the levels of total N-glycans in the plasma and IgG in patients with PTSD and control subjects. We found an association of the *HNF1A-AS1* rs7953249 and the *HNF1A* rs735396 polymorphisms and their haplotype blocks with the levels of complex, highly branched and sialyated plasma N-glycans and core-fucosylated IgG N-glycans with bisecting N-acetylglucosamine (GlcNAc), mostly in patients with PTSD. These polymorphisms were not associated with the methylation of the *HNF1A* gene; however, the levels of triantennary galactosylated and sialyated plasma N-glycans negatively correlated with methylation of the CpG3 site in patients with PTSD. On the other hand, an association between the levels of the two biantennary plasma N-glycans and one IgG core-fucosylated biantennary N-glycan and methylation at the CpG1 or CpG2 sites was observed in the control subjects. These results indicate a possible regulatory role of the investigated *HNF1A* polymorphisms with respect to the abundance of complex plasma N-glycans, which were previously associated with proinflammatory response, which could contribute to the clinical manifestation of PTSD and its comorbidities. Although this study was performed on a relatively high number of age- and sex-matched participants and the *p*-value was corrected for multiple testing, obtained results require additional verification in other ethnic groups and female subjects.

## Figures and Tables

**Table 1:** Distribution of genotypes, alleles and haplotypes of *HNF1A-AS1* rs7953249 and *HNF1A* rs735396 polymorphisms in control subjects and patients with PTSD.

SNP		Control Subjects		Subjects with PTSD		Statistics
		N	%	N	%	
*HNF1A-AS1* rs7953249	AA	86	33.3	113	38.0	χ^2^ = 9.263; df = 2; ***p* = 0.010**
	AG	117	45.3	149	50.2	
	GG	55	21.3	35	11.8	
	A	289	56.0	375	63.1	χ^2^ = 5.830; df = 1; *p* = 0.016
	G	227	44.0	219	36.9	
*HNF1A* rs735396	TT	110	42.6	130	43.8	χ^2^ = 5.605; df = 2; *p* = 0.061
	TC	108	41.9	140	47.1	
	CC	40	15.5	27	9.1	
	T	328	63.6	400	67.3	χ^2^ = 1.589; df = 1; *p* = 0.208
	C	188	36.4	194	32.7	
Haplotype *HNF1A* rs7953249-rs735396	AT	274	53.1	351	59.1	χ^2^ = 6.383; df = 3; *p* = 0.094
	GC	173	33.5	170	28.6	
	GT	54	10.5	49	8.2	
	AC	15	2.9	24	4.0	

Data are presented as the total number and frequency (%), and significant *p*-value (*p* < 0.010) is denoted in bold.

**Table 2:** Methylation at three CpG sites of the *HNF1A* gene in the patients with PTSD and the control subjects subdivided according to the *HNF1A* rs7953249 and rs735396 genotypes, alleles and haplotypes.

Methylation Site	Diagnostic Group	*HNF1A-AS1*		*HNF1A*		Haplotype
		Genetic Model	Allelic Model	Genetic Model	Allelic Model	
CpG1	Control	H = 0.248; df = 2; *p* = 0.883	U = 4713.0; *p* = 0.650	H = 0.006; df = 2; *p* = 0.997	U = 4689.0; *p* = 0.938	H = 0.556; df = 3; *p* = 0.906
	PTSD	H = 0.736; df = 2; *p* = 0.692	U = 4538.5; *p* = 0.831	H = 0.081; df = 2; *p* = 0.960	U = 4129.5; *p* = 0.871	H = 2.521; df = 3; *p* = 0.472
CpG2	Control	H = 1.647; df = 2; *p* = 0.439	U = 4405.0; *p* = 0.223	H = 1.318; df = 2; *p* = 0.517	U = 4372.0; *p* = 0.379	H = 0.181; df = 3; *p* = 0.981
	PTSD	H = 0.007; df = 2; *p* = 0.997	U = 4592.5; *p* = 0.941	H = 0.467; df = 2; *p* = 0.792	U = 4184.5; *p* = 0.989	H = 4.866; df = 3; *p* = 0.182
CpG3	Control	H = 2.156; df = 2; *p* = 0.340	U = 4309.0; *p* = 0.145	H = 3.198; df = 2; *p* = 0.202	U = 4022.0; *p* = 0.078	H = 3.437; df = 3; *p* = 0.329
	PTSD	H = 0.661; df = 2; *p* = 0.719	U = 4357.5; *p* = 0.498	H = 0.692; df = 2; *p* = 0.708	U = 4105.5; *p* = 0.821	H = 1.319; df = 3; *p* = 0.725

Data were analyzed by Kruskal–Wallis test or Mann–Whitney test. df, degrees of freedom; H, Kruskal–Wallis test value; U, Mann–Whitney test value.

**Table 3:** Correlations between methylation at 3 CpG sites of the *HNF1A* gene and the plasma and IgG N-glycan levels.

Glycan Peak	Diagnostic Group	Methylation Site		
		CpG1	CpG2	CpG3
		Spearman’s rho coefficient (*p* value)	Spearman’s rho coefficient (*p* value)	Spearman’s rho coefficient (*p* value)
GP10 (FA2G2)	Control	−0.134 (0.188)	−0.333 (**0.001**)	−0.187 (0.064)
	PTSD	−0.132 (0.195)	−0.033 (0.748)	−0.028 (0.785)
GP14 (A2G2S1)	Control	0.259 (**0.010**)	0.179 (0.076)	0.093 (0.360)
	PTSD	−0.211 (0.037)	−0.220 (0.030)	−0.174 (0.086)
GP21 (A2BG2S2)	Control	0.057 (0.574)	0.079 (0.440)	0.124 (0.220)
	PTSD	−0.271 (**0.007**)	−0.239 (0.018)	−0.251 (0.013)
GP24 (A3G3S2)	Control	−0.029 (0.778)	−0.009 (0.927)	0.089 (0.381)
	PTSD	−0.049 (0.632)	−0.234 (0.020)	−0.276 (**0.006**)
GP26 (A3G3S2)	Control	−0.056 (0.582)	0.051 (0.613)	0.029 (0.779)
	PTSD	−0.101 (0.321)	−0.254 (0.012)	−0.283 (**0.005**)
GP30 (A3G3S3)	Control	−0.051 (0.618)	−0.050 (0.626)	0.044 (0.663)
	PTSD	−0.047 (0.648)	−0.238 (0.018)	−0.270 (**0.007**)
GP31 (FA3G3S3)	Control	−0.017 (0.864)	0.033 (0.746)	−0.034 (0.740)
	PTSD	−0.119 (0.244)	−0.254 (0.012)	−0.258 (**0.010**)
IgGP14 (FA2G2)	Control	−0.070 (0.489)	−0.264 (**0.008**)	−0.163 (0.108)
	PTSD	−0.214 (0.034)	−0.105 (0.304)	−0.017 (0.869)

Data are presented as Spearman’s rho coefficient. Significant *p*-values (*p* < 0.010) are denoted in bold.

**Table 4:** Associations between the *HNF1A-AS1* rs7953249 and the *HNF1A* rs735396 polymorphisms and the N-glycans in control subjects and patients with PTSD.

Glycan Peak	Diagnostic Group	*HNF1A AS1*		*HNF1A*		Haplotype
		Genetic Model	Allelic Model	Genetic Model	Allelic Model	
GP20 (A2G2S2)	Control	H = 4.337; df = 2; *p* = 0.114	U = 29,274.5; *p* = 0.036	H = 7.421; df = 2; *p* = 0.024	U = 26,244.0; ***p* = 0.005**	H = 8.145; df = 3; *p* = 0.043
	PTSD	H = 2.353; df = 2; *p* = 0.308	U = 39,466.5; *p* = 0.429	H = 2.832; df = 2; *p* = 0.243	U = 36,330.5; *p* = 0.190	H = 3.366; df = 3; *p* = 0.339
GP24 (A3G3S2)	Control	H = 4.569; df = 2; *p* = 0.102	U = 29,452.5; *p* = 0.046	H = 6.657; df = 2; *p* = 0.036	U = 26,502.0; ***p* = 0.008**	H = 7.072; df = 3; *p* = 0.070
	PTSD	H = 6.779; df = 2; *p* = 0.034	U = 36,322.5; *p* = 0.019	H = 3.704; df = 2; *p* = 0.157	U = 36,850.5; *p* = 0.296	H = 11.942; df = 3; ***p* = 0.008**
GP27 (A3G3S3)	Control	H = 3.248; df = 2; *p* = 0.197	U = 29,824.5; *p* = 0.077	H = 4.628; df = 2; *p* = 0.099	U = 27,360.0; *p* = 0.033	H = 5.798; df = 3; *p* = 0.122
	PTSD	H = 12.838; df = 2; ***p* = 0.002**	U = 34,190.5; ***p* = 0.001**	H = 12.259; df = 2; ***p* = 0.002**	U = 32,402.5; ***p* = 0.001**	H = 13.673; df = 3; ***p* = 0.003**
GP28 (A3G3S3)	Control	H = 3.105; df = 2; *p* = 0.212	U = 30,204.5; *p* = 0.122	H = 6.085; df = 2; *p* = 0.048	U = 26,972.0; *p* = 0.018	H = 5.716; df = 3; *p* = 0.126
	PTSD	H = 12.309; df = 2; ***p* = 0.002**	U = 34,460.5; ***p* = 0.001**	H = 7.080; df = 2; *p* = 0.029	U = 34,596.5; *p* = 0.028	H = 12.275; df = 3; ***p* = 0.006**
GP30 (A3G3S3)	Control	H = 3.247; df = 2; *p* = 0.197	U = 30,194.5; *p* = 0.121	H = 3.798; df = 2; *p* = 0.150	U = 27,584.0; *p* = 0.046	H = 4.169; df = 3; *p* = 0.244
	PTSD	H = 14.029; df = 2; ***p* = 0.001**	U = 34,054.5; ***p* = 0.001**	H = 9.678; df = 2; ***p* = 0.008**	U = 33,452.5; ***p* = 0.006**	H = 16.311; df = 3; ***p* = 0.001**
GP33 (A4G4S3)	Control	H = 3.702; df = 2; *p* = 0.157	U = 29,734.5; *p* = 0.068	H = 6.904; df = 2; *p* = 0.032	U = 26,502.0; ***p* = 0.008**	H = 8.216; df = 3; *p* = 0.042
	PTSD	H = 8.399; df = 2; *p* = 0.015	U = 35,444.5; ***p* = 0.005**	H = 8.925; df = 2; *p* = 0.012	U = 33,438.5; ***p* = 0.005**	H = 9.770; df = 3; *p* = 0.021
GP35 (A4F1G3S3)	Control	H = 2.617; df = 2; *p* = 0.270	U = 30,150.5; *p* = 0.115	H = 7.175; df = 2; *p* = 0.028	U = 26,310.0; ***p* = 0.006**	H = 8.900; df = 3; *p* = 0.031
	PTSD	H = 6.810; df = 2; *p* = 0.033	U = 36,044.5; *p* = 0.013	H = 11.234; df = 2; ***p* = 0.004**	U = 33,032.5; ***p* = 0.003**	H = 10.520; df = 3; *p* = 0.015
GP37 (A4G4S4)	Control	H = 2.004; df = 2; *p* = 0.367	U = 30,952.5; *p* = 0.271	H = 5.357; df = 2; *p* = 0.069	U = 27,982.0; *p* = 0.080	H = 3.184; df = 3; *p* = 0.364
	PTSD	H = 11.885; df = 2; ***p* = 0.003**	U = 34,992.5; ***p* = 0.003**	H = 3.600; df = 2; *p* = 0.165	U = 35,862.5; *p* = 0.122	H = 10.956; df = 3; *p* = 0.012
GP39 (A4F1G4S4)	Control	H = 1.513; df = 2; *p* = 0.469	U = 30,694.5; *p* = 0.210	H = 3.400; df = 2; *p* = 0.183	U = 27,696.0; *p* = 0.054	H = 3.873; df = 3; *p* = 0.276
	PTSD	H = 4.786; df = 2; *p* = 0.091	U = 36,894.5; *p* = 0.039	H = 10.322; df = 2; ***p* = 0.006**	U = 33,270.5; ***p* = 0.004**	H = 9.531; df = 3; *p* = 0.023
IgGP10 (FA2[6]BG1)	Control	H = 1.815; df = 2; *p* = 0.403	U = 32,528.5; *p* = 0.871	H = 10.827; df = 2; ***p* =0.004**	U = 28,494.0; *p* = 0.151	H = 5.306; df = 3; *p* = 0.151
	PTSD	H = 10.327; df = 2; ***p* = 0.006**	U = 40,734.5; *p* = 0.871	H = 5.342; df = 2; *p* = 0.069	U = 36,724.5; *p* = 0.267	H = 3.074; df = 3; *p* = 0.380
IgGP11 (FA2[3]BG1)	Control	H = 3.903; df = 2; *p* = 0.142	U = 32,194.5; *p* = 0.718	H = 5.492; df = 2; *p* = 0.064	U = 29,614.0; *p* = 0.455	H = 3.665; df = 3; *p* = 0.300
	PTSD	H = 5.028; df = 2; *p* = 0.081	U = 39,974.5; *p* = 0.590	H = 12.236; df = 2; ***p* = 0.002**	U = 36,528.5; *p* = 0.227	H = 2.229; df = 3; *p* = 0.526
IgGP15 (FA2BG2)	Control	H = 1.505; df = 2; *p* = 0.471	U = 30,952.5; *p* = 0.271	H = 3.232; df = 2; *p* = 0.199	U = 28,584.0; *p* = 0.168	H = 2.695; df = 3; *p* = 0.441
	PTSD	H = 11.692; df = 2; ***p* = 0.003**	U = 39,186.5; *p* = 0.353	H = 5.153; df = 2; *p* = 0.076	U = 38,668.5; *p* = 0.905	H = 2.187; df = 3; *p* = 0.535

Data were analyzed by Kruskal–Wallis or Mann–Whitney test. Significant *p*-values (*p* < 0.010) are denoted in bold. df, degrees of freedom; H, Kruskal–Wallis test value; U, Mann–Whitney test value.

## Supplementary Materials

The following supporting information can be downloaded at: https://www.mdpi.com/article/10.3390/genes13061063/s1, Supplementary Table S1. Plasma N-glycan peaks separated by HILIC and their composition as described by Gudelj et al. [17] and Saldova et al. [27]; Supplementary Table S2. IgG N-glycan peaks separated by HILIC-UPLC and their composition as described by Nikolac Perkovic et al. [28]; Supplementary Table S3. Associations of *HNF1A* rs7953249 and rs735396 genotypes, alleles and haplotypes with N-glycan levels in control subjects and patients with PTSD.
