# Supplementary material for: Genetic and Epigenetic Association of Hepatocyte Nuclear Factor-1α with Glycosylation in Post-Traumatic Stress Disorder
Source: Genes (Basel). 2022 Jun 14;13(6):1063. doi: 10.3390/genes13061063 (PMC9223288; doi:10.3390/genes13061063)
Supplement: Supplementary file 1 [file genes-13-01063-s001.zip › genes-1737357-supplementary.pdf]

Supplementary Table S1. The plasma N-glycan peaks separated by the HILIC and their composition as described by Gudelj et al. [17] and Saldova et al. [27].

Glycan peak	Major glycan structure	Description
**GP1**	FA2	agalactosylated
**GP2**	M5, FA2B	high mannose (M5), agalactosylated with bisecting GlcNAc (FA2B)
**GP3**	A2[6]BG1	monogalactosylated with bisecting GlcNAc
**GP4**	FA2[6]G1	monogalactosylated with core fucose
**GP5**	FA2[3]G1	monogalactosylated with core fucose
**GP6**	FA2[6]BG1	monogalactosylated with core fucose and bisecting GlcNAc
**GP7**	M6	high mannose
**GP8**	A2G2	digalactosylated
**GP9**	A2BG2	digalactosylated with bisecting GlcNAc
**GP10**	FA2G2	digalactosylated with core fucose
**GP11**	FA2BG2	digalactosylated with core fucose and bisecting GlcNAc
**GP12**	A2[3]BG1S(3)1	monogalactosylated with bisecting glcnac
**GP13**	FA2[3]G1S(3)1	monogalactosylated and sialylated with core fucose
**GP14**	A2G2S(6)1	digalactosylated and sialylated
**GP15**	A2BG2S1	digalactosylated and sialylated with bisecting glcnac
**GP16**	FA2G2S(6)1	digalactosylated and sialylated with core fucose
**GP17**	FA2BG2S(3)1	digalactosylated and sialylated with bisecting glcnac and core fucose
**GP18**	A2G2S(3,6)2	digalactosylated and disialylated
**GP19**	M9	high mannose
**GP20**	A2G2S(3,6)2	digalactosylated and disialylated
**GP21**	A2BG2S2	digalactosylated and disialylated with bisecting glcnac
**GP22**	FA2G2S(3,6)2	digalactosylated and disialylated with core fucose
**GP23**	FA2BG2S(3,6)2	digalactosylated and disialylated with bisecting glcnac and core fucose
**GP24**	A3G3S(3,6)2	trigalactosylated and disialylated
**GP25**	A3BG3S2	trigalactosylated and disialylated with bisecting glcnac
**GP26**	A3G3S(3,3)2	trigalactosylated and disialylated
**GP27**	A3G3S(3,3,3)3	trigalactosylated and trisialylated
**GP28**	A3G3S(3,3,6)3	trigalactosylated and trisialylated
**GP29**	FA3G3S(3,3,3)3	trigalactosylated and trisialylated with core fucose
**GP30**	A3G3S(3,3,6)3	trigalactosylated and trisialylated
**GP31**	FA3G3S(3,3,6)3	trigalactosylated and trisialylated with core fucose
**GP32**	A3F1G3S(3,3,3)3	trigalactosylated and trisialylated with antennary fucose
**GP33**	A4G4S(3,3,3)3	tetragalactosylated and trisialylated
**GP34**	A4G4S(3,3,6)3	tetragalactosylated and trisialylated
**GP35**	A4F1G3S(3,3,3)3	tetragalactosylated and trisialylated with antennary fucose
**GP36**	A4G4S(3,3,3,3)4	tetragalactosylated and tetrasialylated
**GP37**	A4G4S(3,3,3,6)4	tetragalactosylated and tetrasialylated
**GP38**	A4G4S(3,6,6,6)4	tetragalactosylated and tetrasialylated
**GP39**	A4F1G4S(3,3,3,6)4	tetragalactosylated and etrasialylated with antennary fucose

Structure abbreviations: all N-glycans have two core N-acetylglucosamines (GlcNAcs); F at the start of the abbreviation indicates a core fucose α1-6 linked to the inner GlcNAc; Mx, number (x) of mannose on core GlcNAcs; Ax, number of antenna (GlcNAc) on trimannosyl core; B, bisecting GlcNAc linked β1-4 to β1-3 mannose; Gx, number (x) of β1-4 linked galactose on antenna; [3]G1 and [6]G1 indicates that the galactose is on the antenna of the α1-3 or α1-6 mannose; F(x), number (x) of fucose linked α1-3 to antenna GlcNAc; Sx, number (x) of sialic acids linked to galactose; the numbers 3 or 6 or in parentheses after S indicate whether the sialic acid is in an α2-3 or α2-6 linkage. If there is no linkage number, the exact link is unknown.

Supplementary Table S2. The IgG N-glycan peaks separated by the HILIC-UPLC and their composition as described by Nikolac Perkovic et al. [28]

Glycan peak	Major glycan structure	Description
**IgGP1**	FA1	agalactosylated with core fucose
**IgGP2**	A2	agalactosylated
**IgGP3**	A2B	agalactosylated with bisecting GlcNAc
**IgGP4**	FA2	agalactosylated with core fucose
**IgGP5**	M5	high mannose
**IgGP6**	FA2B	agalactosylated with core fucose and bisecting GlcNAc
**IgGP7**	A2G1	monogalactosylated
**IgGP8**	FA2[6]G1	monogalactosylated with core fucose
**IgGP9**	FA2[3]G1	monogalactosylated with core fucose
**IgGP10**	FA2[6]BG1	monogalactosylated with core fucose and bisecting GlcNAc
**IgGP11**	FA2[3]BG1	monogalactosylated with core fucose and bisecting GlcNAc
**IgGP12**	A2G2	digalactosylated
**IgGP13**	A2BG2	digalactosylated with bisecting GlcNAc
**IgGP14**	FA2G2	digalactosylated with core fucose
**IgGP15**	FA2BG2	digalactosylated with core fucose and bisecting GlcNAc
**IgGP16**	FA2G1S1	monogalactosylated and sialyated with core fucose
**IgGP17**	A2G2S1	digalactosylated and sialyated
**IgGP18**	FA2G2S1	digalactosylated and sialyated with core fucose
**IgGP19**	FA2BG2S1	digalactosylated and sialyated with core fucose and bisecting GlcNAc
**IgGP20**	n.d.	structure not determined
**IgGP21**	A2G2S2	digalactosylated and disialyated
**IgGP22**	A2BG2S2	digalactosylated and disialyated with bisecting GlcNAc
**IgGP23**	FA2G2S2	digalactosylated and disialyated with core fucose
**IgGP24**	FA2BG2S2	digalactosylated and disialyated with core fucose and bisecting GlcNAc

Structure abbreviations: all N-glycans have two core N-acetylglucosamines (GlcNAcs); F at the start of the abbreviation indicates a core fucose α1-6 linked to the inner GlcNAc; Mx, number (x) of mannose on core GlcNAcs; Ax, number of antenna (GlcNAc) on trimannosyl core; B, bisecting GlcNAc linked β1-4 to β1-3 mannose; Gx, number (x) of β1-4 linked galactose on antenna; [3]G1 and [6]G1 indicates that the galactose is on the antenna of the α1-3 or α1-6 mannose; F(x), number (x) of fucose linked α1-3 to antenna GlcNAc; Sx, number (x) of sialic acids linked to galactose; the numbers 3 or 6 or in parentheses after S indicate whether the sialic acid is in an α2-3 or α2-6 linkage. If there is no linkage number, the exact link is unknown.

Supplementary Table S3. The associations of the *HNF1A* rs7953249 and rs735396 genotypes, alleles and haplotypes with the N-glycan levels in the control subjects and the patients with PTSD.

Glycan peak		*HNF1A AS1*			*HNF1A*			Haplotype	
		**Control subjects**	**Subjects with PTSD**		**Control subjects**	**Subjects with PTSD**		**Control subjects**	**Subjects with PTSD**
**GP20**	**AA**	0.665 (-1.258; 2.098)	0.495 (-1.385; 1.834)	**TT**	0.633 (-1.022; 1.975)	0.413 (-0.936; 1.834)			
	**AG**	0.082 (-1.492; 1.350)	-0.164 (-1.728; 1.405)	**TC**	0.089 (-1.493; 1.236)	-0.095 (-1.866; 1.547)	**AT**	0.397 (-1.286; 1.846)	0.230 (-1.493; 1.754)
	**GG**	-0.065 (-1.677; 1.100)	0.329 (-1.751; 2.309)	**CC**	-0.731 (-2.394; 0.555)	0.329 (-2.082; 1.673)	**GC**	-0.220 (-1.677; 1.083)	0.105 (-1.751; 1.579)
		H=4.337; df=2; p=0.114	H=2.353; df=2; p=0.308		H=7.421; df=2; p=0.024	H=2.832; df=2; p=0.243	**GT**	0.633 (-0.760; 1.563)	-0.067 (-1.481; 1.447)
	**A**	0.406 (-1.309; 1.809)	0.191 (-1.519; 1.712)	**T**	0.471 (-1.258; 1.788)	0.191 (-1.493; 1.754)	**AC**	0.472 (-2.087; 1.382)	-1.221 (-2.411; 1.622)
	**G**	0.000 (-1.494; 1.307)	0.103 (-1.751; 1.579)	**C**	-0.182 (-1.728; 1.086)	0.032 (-1.885; 1.579)		H=8.145; df=3; p=0.043	H=3.366; df=3; p=0.339
		U=29274.5; p=0.036	U=39466.5; p=0.429		U=26244.0; **p=0.005**	U=36330.5; p=0.190			
**GP24**	**AA**	-0.114 (-0.340; 0.219)	-0.078 (-0.306; 0.224)	**TT**	-0.123 (-0.340; 0.171)	-0.043 (-0.292; 0.246)			
	**AG**	-0.092 (-0.297; 0.202)	0.068 (-0.241; 0.367)	**TC**	-0.052 (-0.297; 0.267)	0.075 (-0.229; 0.365)	**AT**	-0.107 (-0.324; 0.207)	-0.016 (-0.271; 0.277)
	**GG**	0.057 (-0.223; 0.330)	0.046 (-0.183; 0.322)	**CC**	0.072 (-0.094; 0.261)	0.016 (-0.323; 0.199)	**GC**	0.028 (-0.242; 0.264)	0.075 (-0.213; 0.356)
		H=4.569; df=2; p=0.102	H=6.779; df=2; p=0.034		H=6.657; df=2; p=0.036	H=3.704; df=2; p=0.157	**GT**	-0.107 (-0.336; 0.260)	0.000 (-0.255; 0.317)
	**A**	-0.098 (-0.315; 0.215)	-0.027 (-0.292; 0.263)	**T**	-0.107 (-0.324; 0.211)	-0.015 (-0.271; 0.286)	**AC**	0.000 (-0.300; 0.354)	-0.239 (-0.378; 0.094)
	**G**	-0.002 (-0.270; 0.264)	0.053 (-0.224; 0.354)	**C**	0.022 (-0.245; 0.265)	0.034 (-0.242; 0.322)		H=7.072; df=3; p=0.070	H=11.942; df=3; **p=0.008**
		U=29452.5; p=0.046	U=36322.5; p=0.019		U=26502.0; **p=0.008**	U=36850.5; p=0.296			
**GP27**	**AA**	-0.035 (-0.245; 0.137)	0.137 (-0.101; 0.313)	**TT**	-0.047 (-0.245; 0.137)	0.110 (-0.126; 0.277)			
	**AG**	-0.071 (-0.248; 0.104)	0.030 (-0.195; 0.180)	**TC**	-0.069 (-0.267; 0.104)	0.032 (-0.185; 0.186)	**AT**	-0.049 (-0.245; 0.116)	0.081 (-0.132; 0.268)
	**GG**	-0.167 (-0.355; 0.142)	-0.094 (-0.245; 0.142)	**CC**	-0.179 (-0.371; 0.079)	-0.146 (-0.338; 0.137)	**GC**	-0.134 (-0.314; 0.098)	-0.045 (-0.240; 0.156)
		H=3.248; df=2; p=0.197	H=12.838; df=2; **p=0.002**		H=4.628; df=2; p=0.099	H=12.259; df=2; **p=0.002**	**GT**	-0.071 (-0.255; 0.172)	0.047 (-0.184; 0.222)
	**A**	-0.045 (-0.245; 0.114)	0.082 (-0.138; 0.272)	**T**	-0.049 (-0.247; 0.119)	0.069 (-0.140; 0.268)	**AC**	0.019 (-0.314; 0.113)	0.138 (-0.251; 0.330)

	**G**	-0.093 (-0.278; 0.105)	-0.035 (-0.221; 0.176)	**C**	-0.118 (-0.314; 0.099)	-0.035 (-0.245; 0.182)		H=5.798; df=3; p=0.122	H=13.673; df=3; **p=0.003**
		U=29824.5; p=0.077	U=34190.5; **p=0.001**		U=27360.0; p=0.033	U=32402.5; **p=0.001**			
**GP28**	**AA**	-0.028 (-0.110; 0.092)	-0.060 (-0.168; 0.045)	**TT**	-0.033 (-0.120; 0.079)	-0.034 (-0.158; 0.061)			
	**AG**	0.002 (-0.130; 0.094)	-0.001 (-0.101; 0.115)	**TC**	0.008 (-0.130; 0.100)	0.001 (-0.108; 0.128)	**AT**	-0.020 (-0.125; 0.091)	-0.032 (-0.145; 0.090)
	**GG**	0.034 (-0.082; 0.165)	0.030 (-0.089; 0.137)	**CC**	0.046 (-0.014; 0.167)	-0.036 (-0.118; 0.109)	**GC**	0.029 (-0.103; 0.118)	0.004 (-0.100; 0.130)
		H=3.105; df=2; p=0.212	H=12.309; df=2; **p=0.002**		H=6.085; df=2; p=0.048	H=7.080; df=2; p=0.029	**GT**	-0.041 (-0.125; 0.056)	0.019 (-0.110; 0.112)
	**A**	-0.018 (-0.125; 0.092)	-0.035 (-0.147; 0.088)	**T**	-0.022 (-0.125; 0.091)	-0.026 (-0.143; 0.095)	**AC**	0.013 (-0.128; 0.110)	-0.058 (-0.160; 0.019)
	**G**	0.016 (-0.116; 0.110)	0.012 (-0.101; 0.129)	**C**	0.028 (-0.107; 0.114)	-0.007 (-0.110; 0.121)		H=5.716; df=3; p=0.126	H=12.275; df=3; **p=0.006**
		U=30204.5; p=0.122	U=34460.5; **p=0.001**		U=26972.0; p=0.018	U=34596.5; p=0.028			
**GP30**	**AA**	-0.331 (-0.778; 0.711)	-0.421 (-1.113; 0.447)	**TT**	-0.357 (-0.864; 0.453)	-0.276 (-1.076; 0.495)			
	**AG**	-0.182 (-0.910; 0.720)	0.164 (-0.684; 0.790)	**TC**	-0.179 (-0.868; 0.768)	0.178 (-0.721; 0.895)	**AT**	-0.285 (-0.828; 0.711)	-0.142 (-0.874; 0.669)
	**GG**	0.208 (-0.613; 0.834)	0.595 (-0.785; 1.108)	**CC**	0.289 (-0.449; 0.778)	0.191 (-0.674; 0.760)	**GC**	0.170 (-0.675; 0.773)	0.220 (-0.593; 0.993)
		H=3.247; df=2; p=0.197	H=14.029; df=2; **p=0.001**		H=3.798; df=2; p=0.150	H=9.678; df=2; **p=0.008**	**GT**	-0.168 (-0.929; 0.554)	-0.051 (-0.839; 0.851)
	**A**	-0.245 (-0.792; 0.716)	-0.142 (-0.895; 0.664)	**T**	-0.260 (-0.864; 0.683)	-0.142 (-0.874; 0.700)	**AC**	0.370 (-0.611; 0.785)	-0.366 (-1.162; 0.307)
	**G**	0.060 (-0.742; 0.761)	0.211 (-0.689; 0.993)	**C**	0.175 (-0.674; 0.778)	0.187 (-0.699; 0.851)		H=4.169; df=3; p=0.244	H=16.311; df=3; **p=0.001**
		U=30194.5; p=0.121	U=34054.5; **p=0.001**		U=27584.0; p=0.046	U=33452.5; **p=0.006**			
**GP33**	**AA**	-0.136 (-0.900; 0.489)	0.483 (-0.423; 1.110)	**TT**	-0.131 (-0.842; 0.617)	0.397 (-0.541; 1.100)			
	**AG**	-0.200 (-0.937; 0.396)	0.078 (-0.704; 0.857)	**TC**	-0.319 (-0.994; 0.379)	0.051 (-0.638; 0.869)	**AT**	-0.170 (-0.900; 0.463)	0.299 (-0.550; 1.084)
	**GG**	-0.625 (-1.409; 0.322)	-0.268 (-0.972; 0.486)	**CC**	-0.790 (-1.430; 0.172)	-0.675 (-1.350; 0.424)	**GC**	-0.454 (-1.344; 0.322)	-0.154 (-0.941; 0.687)
		H=3.702; df=2; p=0.157	H=8.399; df=2; p=0.015		H=6.904; df=2; p=0.032	H=8.925; df=2; p=0.012	**GT**	-0.177 (-0.734; 0.732)	0.153 (-0.704; 0.934)
	**A**	-0.169 (-0.900; 0.460)	0.335 (-0.585; 1.086)	**T**	-0.171 (-0.856; 0.476)	0.292 (-0.570; 1.060)	**AC**	-0.150 (-0.937; 0.460)	0.453 (-0.787; 1.615)

	**G**	-0.398 (-1.213; 0.345)	-0.089 (-0.868; 0.788)	**C**	-0.423 (-1.323; 0.327)	-0.089 (-0.897; 0.722)		H=8.216; df=3; p=0.042	H=9.770; df=3; p=0.021
		U=29734.5; p=0.068	U=35444.5; **p=0.005**		U=26502.0; **p=0.008**	U=33438.5; **p=0.005**			
	**AA**	-0.041 (-0.094; 0.050)	0.016 (-0.051; 0.118)	**TT**	-0.024 (-0.093; 0.054)	0.016 (-0.065; 0.118)			
	**AG**	-0.034 (-0.125; 0.050)	-0.007 (-0.105; 0.082)	**TC**	-0.054 (-0.131; 0.051)	-0.005 (-0.101; 0.102)	**AT**	-0.039 (-0.105; 0.053)	0.002 (-0.073; 0.114)
	**GG**	-0.062 (-0.161; 0.038)	-0.053 (-0.137; 0.118)	**CC**	-0.090 (-0.165; 0.023)	-0.089 (-0.141; 0.004)	**GC**	-0.071 (-0.157; 0.037)	-0.039 (-0.120; 0.065)
**GP35**		H=2.617; df=2; p=0.270	H=6.810; df=2; p=0.033		H=7.175; df=2; p=0.028	H=11.234; df=2; **p=0.004**	**GT**	-0.010 (-0.098; 0.058)	0.002 (-0.090; 0.133)
	**A**	-0.039 (-0.105; 0.050)	-0.002 (-0.073; 0.114)	**T**	-0.030 (-0.104; 0.053)	0.002 (-0.073; 0.114)	**AC**	-0.020 (-0.125; 0.042)	-0.009 (-0.072; 0.125)
	**G**	-0.056 (-0.147; 0.040)	-0.030 (-0.116; 0.097)	**C**	-0.069 (-0.152; 0.038)	-0.033 (-0.117; 0.091)		H=8.900; df=3; p=0.031	H=10.520; df=3; p=0.015
		U=30150.5; p=0.115	U=36044.5; p=0.013		U=26310.0; **p=0.006**	U=33032.5; **p=0.003**			
	**AA**	-0.046 (-0.104; 0.058)	-0.049 (-0.109; 0.043)	**TT**	-0.046 (-0.114; 0.064)	-0.034 (-0.090; 0.064)			
	**AG**	-0.042 (-0.114; 0.054)	0.011 (-0.081; 0.123)	**TC**	-0.042 (-0.112; 0.050)	0.009 (-0.093; 0.118)	**AT**	-0.045 (-0.112; 0.058)	-0.027 (-0.097; 0.070)
	**GG**	-0.014 (-0.084; 0.116)	0.012 (-0.064; 0.127)	**CC**	0.006 (-0.063; 0.154)	-0.001 (-0.109; 0.111)	**GC**	-0.022 (-0.087; 0.086)	0.010 (-0.085; 0.126)
**GP37**		H=2.004; df=2; p=0.367	H=11.885; df=2; **p=0.003**		H=5.357; df=2; p=0.069	H=3.600; df=2; p=0.165	**GT**	-0.045 (-0.125; 0.071)	0.031 (-0.052; 0.123)
	**A**	-0.043 (-0.106; 0.058)	-0.027 (-0.101; 0.070)	**T**	-0.045 (-0.114; 0.059)	-0.020 (-0.093; 0.073)	**AC**	-0.032 (-0.077; 0.149)	-0.036 (-0.180; 0.070)
	**G**	-0.025 (-0.100; 0.083)	0.012 (-0.081; 0.126)	**C**	-0.022 (-0.086; 0.086)	0.009 (-0.093; 0.116)		H=3.184; df=3; p=0.364	H=10.956; df=3; p=0.012
		U=30952.5; p=0.271	U=34992.5; p=0.003		U=27982.0; p=0.080	U=35862.5; p=0.122			
	**AA**	-0.130 (-0.316; 0.005)	0.062 (-0.171; 0.302)	**TT**	-0.141 (-0.315; 0.061)	0.064 (-0.171; 0.334)			
	**AG**	-0.169 (-0.394; 0.103)	-0.003 (-0.222; 0.262)	**TC**	-0.182 (-0.405; 0.087)	0.001 (-0.249; 0.255)	**AT**	-0.141 (-0.331; 0.060)	0.029 (-0.211; 0.268)
**GP39**	**GG**	-0.227 (-0.419; 0.031)	-0.143 (-0.342; 0.258)	**CC**	-0.302 (-0.426; -0.030)	-0.151 (-0.425; -0.035)	**GC**	-0.209 (-0.426; 0.046)	-0.087 (-0.274; 0.204)
		H=1.513; df=2; p=0.469	H=4.786; df=2; p=0.091		H=3.400; df=2; p=0.183	H=10.322; df=2; **p=0.006**	**GT**	-0.164 (-0.351; 0.138)	0.061 (-0.255; 0.455)
	**A**	-0.145 (-0.337; 0.060)	0.029 (-0.212; 0.268)	**T**	-0.147 (-0.334; 0.080)	0.034 (-0.222; 0.302)	**AC**	-0.253 (-0.366; 0.205)	-0.078 (-0.281; 0.260)

	**G**	-0.191<br>(-0.401; 0.061)	-0.059<br>(-0.256; 0.259)	**C**	-0.209<br>(-0.422; 0.048)	-0.084<br>(-0.281; 0.241)		H=3.873; df=3;<br>p=0.276	H=9.531; df=3;<br>p=0.023
		U=30694.5;<br>p=0.210	U=36894.5;<br>p=0.039		U=27696.0;<br>p=0.054	U=33270.5;<br>**p=0.004**			
**IgGP10**	**AA**	-0.272<br>(-0.838; 0.381)	-0.126<br>(-0.818; 0.582)	**TT**	-0.187<br>(-0.877; 0.534)	-0.029<br>(-0.659; 0.761)			
	**AG**	-0.011<br>(-0.805; 0.588)	0.052<br>(-0.630; 1.013)	**TC**	0.050<br>(-0.659; 0.597)	-0.068<br>(-0.735; 0.971)	**AT**	-0.149<br>(-0.835; 0.534)	-0.040<br>(-0.701; 0.771)
	**GG**	-0.222<br>(-0.810; 0.464)	-0.382<br>(-1.426; 0.368)	**CC**	-0.562<br>(-1.133; -0.009)	-0.300<br>(-1.426; 0.044)	**GC**	-0.163<br>(-0.812; 0.461)	-0.119<br>(-0.806; 0.863)
		H=1.815; df=2;<br>p=0.403	H=10.327;<br>df=2; **p=0.006**		H=10.827;<br>df=2; **p=0.004**	H=5.342; df=2;<br>p=0.069	**GT**	0.258<br>(-0.599; 0.859)	0.132<br>(-0.860; 0.930)
	**A**	-0.153<br>(-0.835; 0.519)	-0.062<br>(-0.722; 0.771)	**T**	-0.099<br>(-0.828; 0.583)	-0.030<br>(-0.702; 0.863)	**AC**	-0.558<br>(-0.818; -0.004)	-0.330<br>(-1.086; 0.415)
	**G**	-0.113<br>(-0.810; 0.567)	-0.097<br>(-0.808; 0.889)	**C**	-0.210<br>(-0.815; 0.447)	-0.144<br>(-0.827; 0.829)		H=5.306; df=3;<br>p=0.151	H=3.074; df=3;<br>p=0.380
		U=32528.5;<br>p=0.871	U=40734.5;<br>p=0.871		U=28494.0;<br>p=0.151	U=36724.5;<br>p=0.267			
**IgGP11**	**AA**	-0.038<br>(-0.101; 0.034)	0.002<br>(-0.083; 0.074)	**TT**	-0.030<br>(-0.098; 0.064)	0.000<br>(-0.082; 0.082)			
	**AG**	0.007<br>(-0.080; 0.070)	0.014<br>(-0.076; 0.109)	**TC**	0.009<br>(-0.071; 0.067)	0.017<br>(-0.066; 0.110)	**AT**	-0.023<br>(-0.094; 0.059)	0.007<br>(-0.081; 0.087)
	**GG**	-0.024<br>(-0.107; 0.029)	-0.068<br>(-0.103; 0.064)	**CC**	-0.052<br>(-0.107; 0.026)	-0.086<br>(-0.125; -0.038)	**GC**	-0.023<br>(-0.097; 0.049)	-0.027<br>(-0.097; 0.082)
		H=3.903; df=2;<br>p=0.142	H=5.028; df=2;<br>p=0.081		H=5.492; df=2;<br>p=0.064	H=12.236;<br>df=2; **p=0.002**	**GT**	0.003<br>(-0.073; 0.087)	-0.002<br>(-0.080; 0.115)
	**A**	-0.023<br>(-0.094; 0.058)	0.004<br>(-0.082; 0.087)	**T**	-0.018<br>(-0.092; 0.064)	0.007<br>(-0.081; 0.093)	**AC**	0.007<br>(-0.075; 0.049)	-0.019<br>(-0.111; 0.057)
	**G**	-0.014<br>(-0.086; 0.057)	-0.024<br>(-0.088; 0.090)	**C**	-0.023<br>(-0.087; 0.049)	-0.026<br>(-0.098; 0.082)		H=3.665; df=3;<br>p=0.300	H=2.229; df=3;<br>p=0.526
		U=32194.5;<br>p=0.718	U=39974.5;<br>p=0.590		U=29614.0;<br>p=0.455	U=36528.5;<br>p=0.227			
**IgGP15**	**AA**	-0.037<br>(-0.218; 0.254)	-0.074<br>(-0.306; 0.126)	**TT**	-0.034<br>(-0.218; 0.254)	-0.046<br>(-0.295; 0.197)			
	**AG**	-0.015<br>(-0.185; 0.160)	0.057<br>(-0.178; 0.320)	**TC**	-0.001<br>(-0.188; 0.168)	0.036<br>(-0.177; 0.219)	**AT**	-0.030<br>(-0.200; 0.243)	-0.021<br>(-0.269; 0.187)
	**GG**	-0.070<br>(-0.244; 0.174)	-0.146<br>(-0.417; 0.141)	**CC**	-0.108<br>(-0.264; 0.070)	-0.154<br>(-0.454; 0.142)	**GC**	-0.026<br>(-0.210; 0.154)	0.011<br>(-0.247; 0.159)
		H=1.505; df=2;<br>p=0.471	H=11.692;<br>df=2; **p=0.003**		H=3.232; df=2;<br>p=0.199	H=5.153; df=2;<br>p=0.076	**GT**	-0.046<br>(-0.235; 0.228)	0.028<br>(-0.197; 0.235)
	**A**	-0.031<br>(-0.200; 0.227)	-0.032<br>(-0.272; 0.167)	**T**	-0.031<br>(-0.201; 0.233)	-0.021<br>(-0.247; 0.197)	**AC**	-0.096<br>(-0.292; 0.048)	-0.135<br>(-0.293; 0.086)

	**G**	-0.032 (-0.212; 0.165)	0.011 (-0.247; 0.229)	**C**	-0.032 (-0.214; 0.151)	0.007 (-0.269; 0.158)		H=2.695; df=3; p=0.441	H=2.187; df=3; p=0.535
		U=30952.5; p=0.271	U=39186.5; p=0.353		U=28584.0; p=0.168	U=38668.5; p=0.905			

The data are presented as age-adjusted percentage of total N-glycan peak area (median and interquartile range) analyzed using Kruskal-Wallis or Mann-Whitney test statistics. Significant p-values ($p<0.010$) are denoted in bold. df-degrees of freedom; H-Kruskal-Wallis test value; U-Mann-Whitney test value
